# MicroRNAs Present in Malignant Pleural Fluid Increase the Migration of Normal Mesothelial Cells In Vitro and May Help Discriminate between Benign and Malignant Effusions

**DOI:** 10.3390/ijms241814022

**Published:** 2023-09-13

**Authors:** Marta Marqués, Mariona Pont, Iván Hidalgo, Maria Alba Sorolla, Eva Parisi, Antonieta Salud, Anabel Sorolla, José M. Porcel

**Affiliations:** 1 Research Group of Cancer Biomarkers, Lleida Institute for Biomedical Research Dr. Pifarré Foundation (IRBLleida), Avda Alcalde Rovira Roure 80, 25198 Lleida, Spain; martamartasun@hotmail.com (M.M.); mpont@irblleida.cat (M.P.); ihidalgo@irblleida.cat (I.H.); msorolla@irblleida.cat (M.A.S.); eparisi@irblleida.cat (E.P.); asaluds@hotmail.com (A.S.); asorolla@irblleida.cat (A.S.); 2 Department of Medical Oncology, Arnau de Vilanova University Hospital, Avda Alcalde Rovira Roure 80, 25198 Lleida, Spain; 3 Pleural Medicine and Clinical Ultrasound Unit, Department of Internal Medicine, Arnau de Vilanova University Hospital, Avda Alcalde Rovira Roure 80, 25198 Lleida, Spain

**Keywords:** malignant pleural effusion, microRNA, cell proliferation, cell migration, cell viability

## Abstract

The sensitivity of pleural fluid (PF) analyses for the diagnosis of malignant pleural effusions (MPEs) is low to moderate. Knowledge about the pathobiology and molecular characteristics of this condition is limited. In this study, the crosstalk between stromal cells and tumor cells was investigated in vitro in order to reveal factors that are present in PF which can mediate MPE formation and aid in discriminating between benign and malignant etiologies. Eighteen PF samples, in different proportions, were exposed in vitro to mesothelial MeT-5A cells to determine the biological effects on these cells. Treatment of normal mesothelial MeT-5A cells with malignant PF increased cell viability, proliferation, and migration, and activated different survival-related signaling pathways. We identified differentially expressed miRNAs in PF samples that could be responsible for these changes. Consistently, bioinformatics analysis revealed an enrichment of the discovered miRNAs in migration-related processes. Notably, the abundance of three miRNAs (miR-141-3p, miR-203a-3, and miR-200c-3p) correctly classified MPEs with false-negative cytological examination results, indicating the potential of these molecules for improving diagnosis. Malignant PF produces phenotypic and functional changes in normal mesothelial cells. These changes are partly mediated by certain miRNAs, which, in turn, could serve to differentiate malignant from benign effusions.

## 1. Introduction

Malignant pleural effusion (MPE) is a serious condition in which fluid accumulates in the pleural space due to increased vascular permeability combined with lymphatic vessel obstruction by parietal pleural implants [1]. MPE usually indicates metastasis of an already existing primary tumor [1]. A study of 840 MPEs showed that the most common primary tumors that metastasize to the pleura were lung cancer (37%), breast cancer (16%), cancer of unknown origin (10%), hematologic malignancies (10%), gastrointestinal tumors (8%), and ovarian cancer (7%) [2].

It is widely accepted that during the metastatic process, crosstalk between tumoral cells and stromal cells establishes the metastatic niche and supports tissue invasion [3,4]. Regarding MPEs, previous works have indicated that the nuclear factor kappa B signaling pathway participates in the disease process [5] through the action of tumor necrosis factor [6]. Other molecules that are involved in pleural fluid (PF) formation include signal transducer and activator of transcription 3 (STAT3), vascular endothelial growth factor (VEGF) [6,7], transforming growth factor β (TGF-β) [8], interleukin (IL)-10 [9], and IL-6 [10]. However, the precise mechanism underlying the biopathogenesis of MPE is not yet fully understood.

MicroRNAs (miRNAs) are a group of non-coding RNAs comprising approximately 22 nucleotides, which act as regulators of post-transcriptional gene expression. It has been demonstrated that they regulate biological processes such as cell proliferation, differentiation, and apoptosis [11]. In recent decades, substantial attention has been given to the role of microRNAs (miRNAs) in the initiation and progression of cancer [12,13], as well as their use as valuable diagnostic and prognostic biomarkers of various malignancies [11,14,15,16], including MPEs [17]. However, evidence demonstrating that miRNAs can regulate the MPE process is rather scarce. In this regard, Zhai et al. showed that miR-7116-5p negatively regulates IL-10 to suppress MPE formation [18]. Another study demonstrated that miR-93 targets angiopoietin2, halting the MPE development process [19]. Moreover, Lv et al. found that miR141 promotes MPE formation by decreasing C-X-C Motif Chemokine Ligand 1 (CXCL1) production and the recruitment of regulatory T cells [20].

In addition to an incompletely understood pathogenesis, the diagnosis of MPE is hampered by the moderate sensitivity of the PF cytological examination (approximately 55%) [21]. For the clinician, it is important to exploit as much information as possible from the PF that is aspirated with a simple thoracentesis, which includes liquid biopsy (e.g., miRNAs), instead of indicating more invasive diagnostic methods such as pleural biopsies.

The aim of this study was to elucidate the miRNA contents of PF samples from patients with benign and MPEs, as well as their biological effects on pleural mesothelial cells in vitro. Additionally, we aimed to identify a miRNA signature that could be used to discriminate between benign and MPEs.

## 2. Results

### 2.1. Characteristics of the Study Population

PF samples were subjected to biochemical and/or cytological examinations: 6 PF samples (B1–B6) were benign and 12 PF samples were malignant (M1–M12). The patient characteristics, the primary tumor types, and the etiologies of benign pleural effusions are shown in Table 1. Among the patients with MPE, patients M2, M10, M11, and M12 had negative PF cytological examination results and/or pleural biopsies and were diagnosed on clinical grounds.

### 2.2. Effect of Pleural Fluid on the Viability of MeT-5A Cells

**Figure 1:**
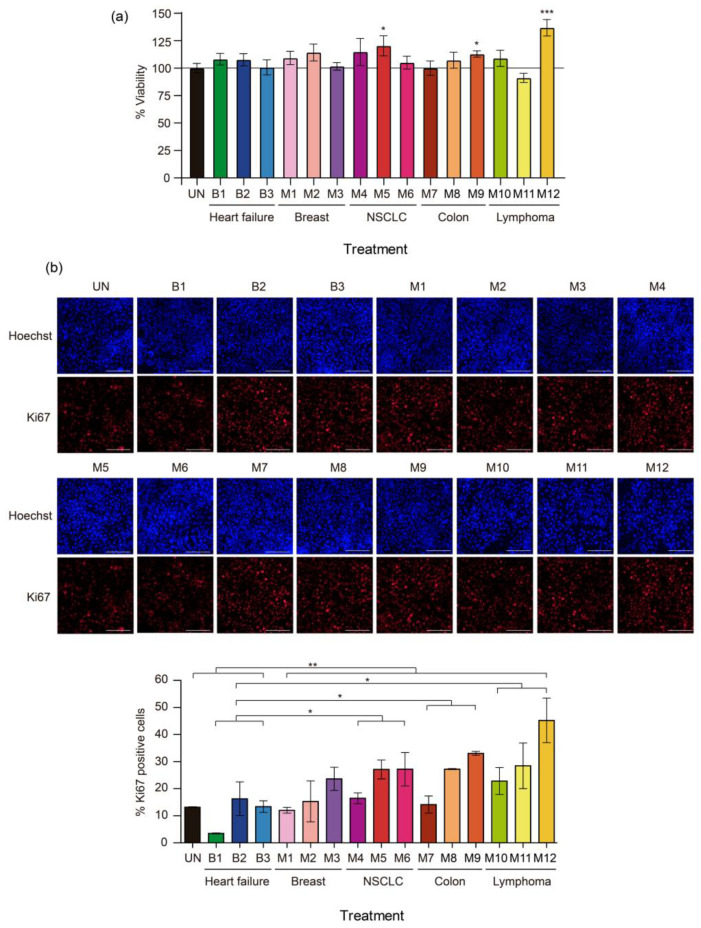
Effects of malignant pleural fluid (PF) on the viability and proliferation of normal mesothelial MeT-5A cells. (**a**) Viability of MeT-5A cells exposed to different PF samples for 24 h. The line indicates 100% viability. (**b**) Representative images of Ki-67 immunofluorescence (red) 24 h after treatment with PF. Each image is shown with the corresponding nuclear staining (Hoechst). The scale bar indicates 200 µm. At the bottom, a graph of Ki-67 quantification shows the percentage of Ki-67-positive cells. The data are presented as the mean ± standard error of the mean of three independent experiments. Differences are considered significant at *p* < 0.05 (*), *p* < 0.05 (**), and *p* < 0.005 (***), as determined by two-tailed Student’s *t*-test. Abbreviations: B, benign; M, malignant; NSCLC, non-small cell lung cancer; UN, untreated condition (control).

The biological effects of PF on the viability of normal mesothelial cells were investigated. The latter were used to explore a tumor–stroma crosstalk mechanism that could influence the formation of an MPE. MeT-5A cells were treated for 24 h with media supplemented with a 10% volume of 15 different PF samples: samples B1–B3 and samples M1–M12. As shown in Figure 1a, all the PF samples increased MeT-5A cell viability to varying degrees, except for samples M7 (colorectal cancer) and M11 (lymphoma), which reduced MeT-5A cell viability compared to the untreated condition (control group). PF samples M5 (non-small cell lung cancer (NSCLC)), M9 (colorectal cancer), and M12 (lymphoma) exerted a strong and significant effect on MeT-5A cell viability, with increments of 16.8% (*p* = 0.041), 10.65% (*p* = 0.032), and 12.73% (*p* = 0.0002), respectively, compared to the untreated condition. However, the miRNAs that were extracted from PF did not recapitulate the changes that occurred after treatment with media supplemented with 10% PF. None of the miRNA extracts were able to increase cell viability (Supplementary Figure S1a). Notably, 10% malignant PF supplementation increased MeT-5A cell viability significantly more than benign PF sample supplementation (*p* = 0.017) and all the corresponding miRNA extracts (*p* < 0.0001).

### 2.3. Effect of Pleural Fluid on the Proliferation of MeT-5A Cells

Most malignant PF samples increased the proliferation of MeT-5A cells but MeT-5A cell proliferation in the benign PF treatment groups remained similar to that in the untreated group; sample B1 was the exception among the benign PF samples, as it significantly decreased MeT-5A cell proliferation (*p* = 0.00057) (Figure 1b). The malignant PF samples that caused more marked increases in the percentage of Ki-67-positive cells compared to the untreated condition were M5, M6, M8, M9, M11, and M12 (204.3%, 209.1%, 206.8%, 252%, 202.1%, and 340.7%, respectively; the difference was significant for M8 (*p* = 0.0002)). PF samples M5, M9, and M12 also induced a non-significant increase in MeT-5A cell viability. As expected, malignant PF samples as a group significantly increased the proliferation of MeT-5A cells compared to benign PF samples (*p* = 0.0046). Specifically, PF samples from NSCLC (M4–M6), colorectal cancer (M7–M9), and lymphoma (M10–M12) caused significant increases in MeT-5A cell proliferation (*p* = 0.014, 0.015, and 0.015, respectively) compared to benign PF samples.

Treatment with miRNAs that were extracted from PF did not reproduce the changes in cell proliferation that were observed when MeT-5A cells were treated with PF-supplemented media. Except for B2, M1, and M5, none of the corresponding miRNAs induced significant changes in the percentage of Ki-67-positive cells (Supplementary Figure S1b). Thus, media supplemented with 10% malignant PF was more effective than media supplemented with benign PF or miRNA equivalents in promoting the proliferation of MeT-5A cells.

### 2.4. Effects of Pleural Fluid on the Migration of MeT-5A Cells

**Figure 2:**
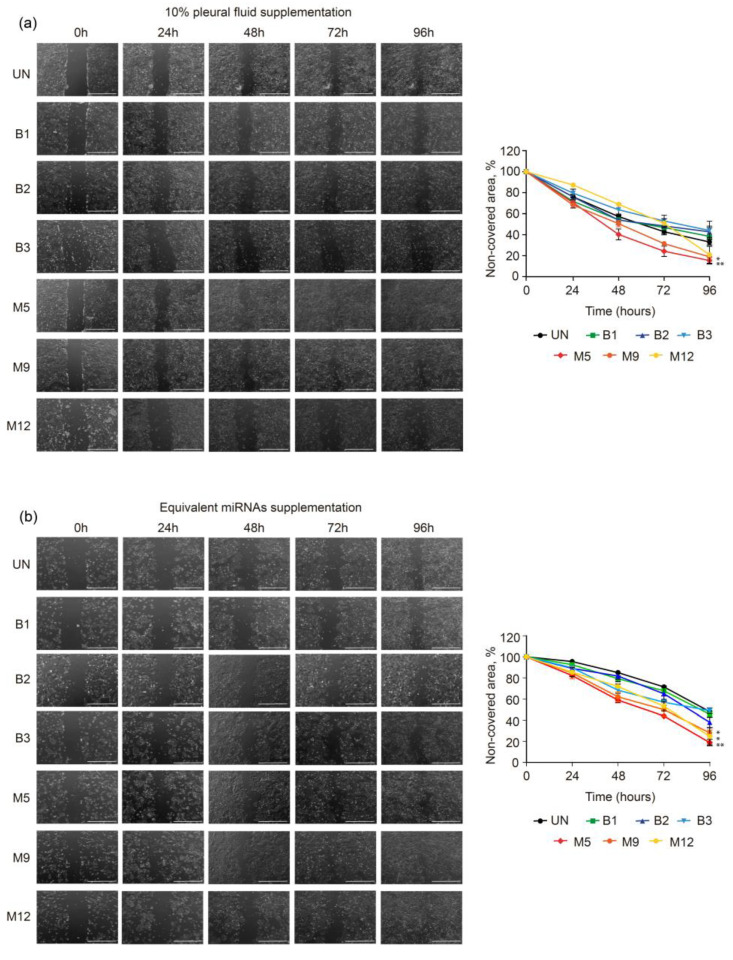
Effects of malignant and benign pleural fluid (PF) on the migration of normal mesothelial MeT-5A cells. Representative brightfield images of MeT-5A cells taken at 0, 24, 48, 72, and 96 h after wounding and treatment with 10% PF (**a**) or equivalent amounts of miRNAs (**b**). The scale bar indicates 1 mm. On the right side, the graphs of the migration assay results are shown, and the data are presented as % cell-free area. The data are presented as the mean ± standard error of the mean of three independent experiments. Differences are considered significant at *p* < 0.05 (*) and *p* < 0.05 (**), as determined by two-tailed Student’s *t*-test. Abbreviations: B, benign; M, malignant; PF, pleural fluid; UN, untreated condition (control).

The rationale of using MeT-5A cells for migration studies is that, due to the interaction and crosstalk between tumor microenvironment and neoplastic cells, these normal mesothelial cells could potentially change their phenotypic characteristics to favor the survival and progression of malignant cells in the PF. The capacity of PF to induce changes in MeT-5A cell migration was examined with wound-healing assays. Both malignant PF and miRNA treatments induced a higher rate of cell migration than benign PF treatment or the untreated condition (Figure 2). Specifically, the degree of wound healing was observed 24 h after 10% PF was added to MeT-5A cell cultures (48 h after seeding, indicated as 48 h in Figure 2), and 40% (*p* = 0.002), 51%, and 69% of the wounded area remained cell-free in the M5, M9, and M12 treatment groups, respectively, compared to the untreated group (57%). When the cells were treated with miRNA samples, the percentage of the wounded area that remained cell-free in the M5, M9, and M12 treatment groups was 59% (*p* = 0.00029), 62% (*p* = 0.0033), and 72% (*p* = 0.0058), respectively, all of which were significantly lower than that in the untreated group (83%). At 48 h post-treatment (indicated as 72 h in Figure 2), the percentage of the wounded area that remained cell-free in the M5, M9, and M12 groups was 24% (*p* = 0.002), 32% (*p* = 0.029), and 51% (*p* = 0.36), respectively, compared to the untreated group (43%). At the same time point, all the corresponding miRNAs significantly improved wound healing, with 44% (*p* = 0.00029), 50% (*p* = 0.014), and 53% (*p* = 0.0082) of the wounded area remaining cell-free, respectively, compared to the untreated group (70%). Finally, at 72 h post-treatment (indicated as 96 h in Figure 2), samples M5, M9, and M12 induced greater cell migration than the untreated condition, with 16% (*p* = 0.00084), 19% (*p* = 0.0065), and 21% of the wounded area remaining cell-free, respectively, while in the untreated group, this value was 33%. Treatment with the miRNA fractions of samples M5, M9, and M12 significantly promoted wound healing, with a smaller percentage of the wounded area remaining cell-free (19%, *p* = 0.00043; 28%, *p* = 0.0021; and 25%, *p* = 0.021, respectively) compared to the untreated group (50%). In summary, media supplementation with 10% PF and the corresponding miRNAs that were extracted from malignant PF were able to increase the migration of MeT-5A cells compared to media supplementation with benign PF.

To complement the cell migration analysis, we investigated the expression of mesenchymal markers by qRT-PCR in MeT-5A cells treated with 10% PF. We observed an increase in the expression of N-Cadherin (*NCAD*) and Vimentin (*VIM*) when MeT-5A cells were treated with sample M9 compared to sample B2. In fact, the expression of *NCAD* and *VIM* remained almost unaltered with sample B2 (Supplementary Figure S2).

### 2.5. Effects of Pleural Fluid on the Phosphorylation of Proteins That Participate in Cell Survival-and Proliferation-Related Signaling Pathways

**Figure 3:**
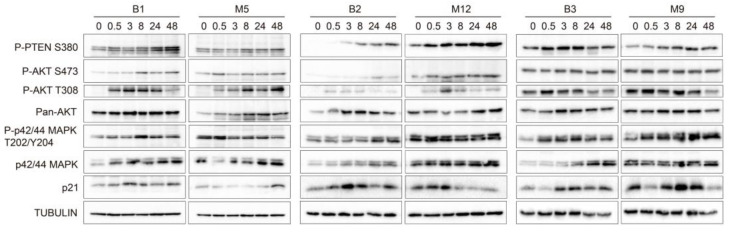
Effects of malignant pleural fluid (PF) on the phosphorylation of different proteins that participate in cell survival- and proliferation-related signaling pathways. Western blot images show whole protein lysates of MeT-5A cells treated with PF samples B1, M5, B2, M12, B3, and M9 for 0, 0.5, 3, 8, 24, and 48 h. The antibodies that were used recognized phosphatase and tensin homolog serine 380 (P-PTEN S380), phospho-AKT serine 473 (P-AKT S473), phospho-AKT threonine 308 (P-AKT T308), pan AKT, phospho-p42/44 MAPK threonine 202/tyrosine 204 (P-p42/44 MAPK T202/Y204), p42/44 MAPK, and p21. Tubulin was used as the control. The Western blots are shown in three panels: B1-M5, B2-M12, and B3-M9. Graphs showing band intensity quantification and the calculated phosphorylated/non-phosphorylated ratio for each protein are shown in Supplementary Figure S3. Abbreviations: B, benign; M, malignant.

The malignant PF samples that caused greater changes in cell viability, proliferation, and migration were selected for this experiment (i.e., M5, M12, and M9). To clarify the signaling pathways that could drive these processes, we investigated changes in the phosphorylation of key proteins involved in two of the most commonly altered signaling pathways in cancer, namely, the AKT and MAPK pathways. Changes in the phosphorylation pattern of the involved proteins indicate activation or deactivation of the pathways. We matched sample M5 with sample B1, sample M12 with sample B2, and sample M9 with sample B3. All the Western blotting experiments were performed using the same protocol. According to the Western blotting results (Figure 3) and the quantification of band intensities (Supplementary Figure S3), malignant PF samples M9 and M12 increased the phosphorylation of P-AKT S473, P-AKT T308, and P-p42/44 MAPK T202/Y204, or these phosphorylation events occurred earlier, compared to the benign PF samples. Similarly, the expression of p42/44 MAPK in MeT-5A cells was increased by malignant PF treatment, while the expression of pan AKT did not appear to change between the benign and malignant PF treatment groups (Figure 3). The expression of P-PTEN S380, which is a marker of a less active and more stable form of PTEN [22], was enhanced when MeT-5A cells were treated with sample M12 compared to sample B2. Treatment of MeT-5A cells with other malignant PF samples (M5 and M9) decreased P-PTEN S380 expression compared to treatment with B1 and B2, respectively, indicating that PTEN was more active but less stable. Finally, p21 expression was reduced by malignant PF treatment compared to benign PF treatment. Overall, it seemed that treatment with malignant PF samples caused a higher phosphorylation of key components of the PI3K/AKT and MAPK signaling pathways and lower p21 expression in MeT-5A cells than treatment with benign PF samples. Additionally, PTEN was less active but more stable after M12 treatment. Next, we determined whether miRNA extracts could exert the same effects on the signaling pathways as 10% PF supplementation. MeT-5A cells were exposed to miRNAs that were extracted from the PF samples that caused the greatest differences when cells were treated with 10% PF supplementation (B2 vs. M12), but we did not observe notable changes in any of the phosphoproteins that participate in the MAPK and PTEN/PI3K/AKT pathways or in p21 expression (Supplementary Figure S4). A signaling pathway diagram with the proteins that were over- and under-expressed in our experiments is shown in Supplementary Figure S5.

### 2.6. Whole Exosome-miRNA Sequencing to Discriminate Benign from Malignant Pleural Fluid Samples

**Figure 4:**
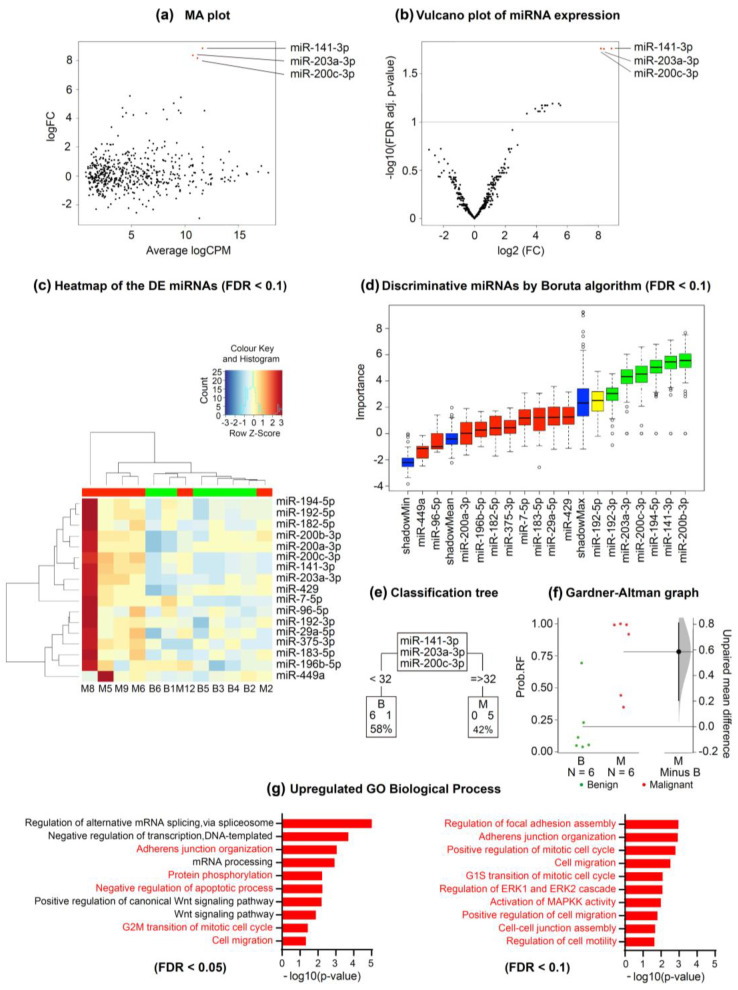
Whole exosome-miRNA sequencing to discriminate benign from malignant pleural fluid samples. (**a**) Log ratio and log mean average plot showing the relationship between the logarithm of fold-change and average logarithm counts per million across the miRNAs analyzed. (**b**) Volcano plot showing the relationship between minus logarithm10 of FDR adjusted *p* value and logarithm2 of FC across the miRNAs analyzed. The red dots represent the significantly upregulated miRNAs in MPEs with respect to benign samples. (**c**) Heatmap showing the DE miRNAs between benign and MPE samples. (**d**) Graph showing the discriminative miRNAs between benign and MPEs according to the Boruta algorithm (FDR < 0.1). Importance means contribution. (**e**) Classification trees for each of the significantly upregulated miRNAs depending on their CPM. The lower boxes represent the number of PEs that were classified as benign or malignant according to miRNA quantification. (**f**) Gardner–Altman graph showing the predicted probability of malignancy and its unpaired mean difference (M minus B). The mean difference and its 95% CI are displayed as a point estimate and a vertical bar, respectively. (**g**) GO analysis for biological processes of the three significantly upregulated miRNAs (FDR < 0.05). The relevant processes are highlighted in red. Abbreviations: B, benign; CPM, counts per million; DE, differentially expressed; FC, fold-change; FDR, false discovery rate; GO, gene ontology; M, malignant; MA, log ratio minus log average; MPE, malignant pleural effusion; PE, pleural effusion; RF, random forest; Wnt, Wingless/Integrated.

Finally, we performed whole exosome-miRNA sequencing on 6 benign and 6 malignant PF samples to identify putative miRNAs that could be responsible for the previously observed biological effects. Among the 2632 investigated miRNAs, we found 3 and 17 that were significantly upregulated in the MPE samples versus the benign PF samples with FDRs < 0.05 and <0.1, respectively (Figure 4a–c). The first three miRNAs were miR-200c-3p, miR-203a-3p, and miR-141-3p. The PF samples that were analyzed were clustered according to the expression of the 17 upregulated miRNAs, and their contribution to discriminating benign and malignant PF samples was arranged and plotted (Figure 4c). The classification trees showed that ˂32 counts per million (CPM) of miR-141-3p or miR-203a-3p was 100% indicative of a benign condition, and all 6 benign PF samples met this criterion. Regarding miR-200c-3p, 5 of the 6 benign PF samples met the preceding threshold. Conversely, ≥32 CPM of miR-141-3p, miR-203a-3p, or miR-200c-3p was indicative of malignancy in 83.3% of cases (5/6 for each miRNA) (Figure 4e). The area under the curve (AUC) of this three-miRNA rule was 0.917 (95% confidence interval [CI] 0.75–1).

Next, we performed a random forest analysis on the expression values of miR-141-3p and miR-203a-3p, and the Gardner–Altman graph showed the out-of-bag predicted probabilities and the estimated mean difference between benign and malignant samples. The 95% CI was based on the sample density distribution that was calculated from a bias-corrected and accelerated bootstrap analysis of 5000 resamples. According to this analysis, the identified two-miRNA signature had high discrimination power for distinguishing benign and MPEs, with a predicted AUC of 0.944 (95% CI 0.778–1) (Figure 4f). For random forest probabilities higher than 0.3, the sensitivity of these miRNAs in discriminating benign PF samples from MPEs was 100% (6/6), and the specificity was 83.3% (5/6). Of note, considering <32 CPM for both miR-141-3p and miR-203-3p, all the benign and malignant PF samples were correctly classified (100% sensitivity and specificity).

In our study, 4 of 12 MPE samples (M2, M10, M11, and M12) had negative cyto-histological pleural examination results, which risked the misclassification of these samples as being benign. However, with the application of the decision trees, all these samples were correctly identified as malignant. The Gene Ontology (GO) analysis showed that the significantly upregulated miRNAs were enriched in several biological processes (Figure 4g). We found 42 significantly associated processes with an FDR < 0.05 and plotted the 10 most relevant processes in Figure 4g.

## 3. Discussion

We found that compared to benign PF samples, MPE samples increased the viability, proliferation, and migration of normal mesothelial MeT-5A cells as well as the phosphorylation of proteins that participate in signaling pathways that are related to survival and proliferation. The effects on cell migration were reproduced by the corresponding miRNA fractions from the PF samples. Moreover, the qRT-PCR analysis revealed an increase in the expression of mesenchymal markers when MeT-5A cells were treated with malignant PFs. Whole exosome-miRNA sequencing disclosed that the upregulation of three miRNAs can accurately discriminate between benign and MPEs. These miRNAs are involved in migration-related processes, providing information about the tumor–stromal crosstalk and pathobiology of MPE.

Some studies have shown that malignant PF can support the cellular proliferation of patient-derived malignant mesothelioma cells [23,24] and even elicit a drug protection effect [24]. However, the suggested mechanisms by which PF from MPE reprograms or damages healthy cells in culture, ultimately promoting tumor formation, were not elucidated.

In contrast to our findings, previous articles did not describe significant differences in the proliferation and migration of patient-derived mesothelioma cell cultures, established cultures of MPEs from breast cancer, and lung adenocarcinoma patients, or commercial mesothelioma cell lines treated with different types of PF [23,24]. Cheah et al. observed that MPE samples increased the proliferation of mesothelioma cell lines in a non-significant manner compared to benign PEs, but the experimental conditions (48 h and 30% PF supplementation in culture media) differed from ours [23,24]. Asciak et al. used a smaller number of PF samples, and the lack of figure legends in the publication do not allow the assessment of whether MPE samples induced more proliferation in patient-derived cancer cell cultures than non-MPE samples [23,24].

Furthermore, we observed that PF can alter certain signaling pathways that are implicated in proliferation, survival, and cell cycle progression, with PF samples from MPE patients inducing the greatest effect. To the best of our knowledge, we are the first to identify changes in the phosphorylation of proteins in the PI3K/AKT and MAPK pathways as well as a reduction in the expression of p21, which is a negative regulator of the cell cycle [25], in normal MeT-5A cells after treatment with malignant PF samples. In agreement with our results, a previous study demonstrated that the exposure of cancer cells to malignant PF samples induced epithelial-to-mesenchymal transition (EMT), which is an exacerbated stem cell phenotype, mainly through the activation of the PI3K/AKT/mTOR axis [26]. VEGF, which is secreted by normal mesothelial cells, proved to be relevant for MPE formation, as it increases vascular permeability [27]. In fact, pleural VEGF levels were positively correlated with the MPE volume in vivo when rabbits were stimulated with TGF-β [27]. In addition, treatment of MeT-5A cells with cytokines secreted by peripheral blood mononuclear cells that had been in contact with asbestos resulted in increased MeT-5A cell proliferation [28]. Another study reported that the administration of IL-5 induced MPE formation in syngeneic models of lung adenocarcinoma and colorectal cancer [29]. All these works suggested the involvement of host–tumor cell crosstalk in MPE formation or during tumorigenesis. In brief, MPEs seem to induce changes in mesothelial cells, so that they become tumor-promoting cells or tumor-associated mesothelial cells through tumor–host interactions. During this process, mesothelial cells lose their epithelial phenotype and acquire a mesenchymal phenotype with new enhanced capabilities such as cell migration.

We hypothesized that free circulating miRNAs are good candidates that may be responsible for the phenotypic changes observed in MeT-5A cells, given their relevant implication in cell-to-cell communication [30]. To test our hypothesis, we extracted miRNAs from PF and analyzed whether the PF-induced changes in MeT-5A cell viability, proliferation, and migration could be reproduced. Interestingly, exposure of these cells to the same amount of miRNAs as that found in 10% PF-supplemented medium resulted in similar changes in cell migration, although malignant PF samples enhanced the migration capabilities of MeT-5A cells to the greatest extent. In contrast, extracted miRNAs did not cause significant changes in the viability or proliferation of MeT-5A cells. These findings were supported by transcriptomic data. Additionally, the GO analysis revealed a significant enrichment of miRNA-regulated biological processes that are related to migration, such as adherens junction organization and cell migration [31]. Other processes that were enriched included the Wnt signaling pathway and protein phosphorylation, which are also known to regulate cell migration [32,33]. Notably, the most highly upregulated miRNAs in our study, namely, miR-200c-3p, miR-203a-3p, and miR-141-3p, have been shown to be overrepresented in exosomes derived from the PF of lung adenocarcinoma patients compared to exosomes derived from benign PF [34]. Additionally, extracellular vesicles from other biological matrices, such as supernatants of malignant ascites, had higher levels of miR-200c-3p than benign peritoneal fluid [35]. Both tumor-suppressive and tumor-promoting roles have been described for miR-200c-3p; this molecule suppresses EMT, migration, invasion, and drug resistance [36], and it promotes drug resistance and reduces T-cell infiltration [37,38]. Similarly, to our work, exposure of ovarian cancer spheroids and gastric cancer cells to miR-200c-3p and miR-200c-3p-rich supernatants of malignant ascites, respectively, increased cell migration [35,39]. Moreover, miR-203a-3p can act as a tumor suppressor gene [40] by downregulating insulin growth factor 1 receptor [41] and thrombospondin 2 expression [42], suppressing metastasis and invasion [43,44], and enhancing drug sensitivity [36]. It can also play a role as an oncogene by promoting drug resistance, proliferation, migration, and invasion [45,46,47]. In one study, miR-203a-3p was found to be the most highly expressed miRNA in extravesicular vesicles that were isolated from the pulmonary tumor-draining vein of patients with NSCLC, and this molecule was associated with tumor relapse [48]. Finally, miR-141-3p also plays both tumor-suppressive and tumor-supportive roles. For instance, it has been reported to promote proliferation, angiogenesis, metastasis, and drug resistance [49,50,51,52], but also to reduce proliferation, migration, invasion, and autophagy [53,54,55,56].

By applying the number of counts of the new three-miRNA signature, we were able to correctly classify MPEs that were initially misdiagnosed due to false-negative PF cytological/histological examination results; the use of this signature in the clinic would allow further invasive diagnostic testing to be prevented. Notably, considering a threshold value < 32 CPM for both miR-141-3p and miR-203-3p, all the benign and malignant PF samples were correctly classified. This highlights the diagnostic potential of this miRNA signature, which needs to be formally tested in larger clinical studies.

One limitation of this study is the relatively low number of PF samples that were used for miRNA profile mapping. However, despite having a limited sample size, we were able to identify a miRNA signature that discriminated between benign and malignant conditions. This, together with the in vitro experiments, helped us to better understand the biological effects (e.g., proliferation and migration) exerted by PF and the miRNAs they contain on normal mesothelial MeT-5A cells. Another limitation was the impossibility of studying the significance of all potential PF biomarkers, such as carbohydrates, proteins, lipids, DNA, metabolites, or other RNA species, some of which may also play a role in MPE initiation.

## 4. Materials and Methods

### 4.1. Cell Lines and Culture Conditions

The MeT-5A cell line, which is an epithelial cell line derived from the mesothelium of non-cancerous PF of patients, was purchased from the American Type Culture Collection (ID: CRL-944 TM). MeT-5A cells were transfected with the pRSV-T plasmid (an SV40 ori-construct containing the SV40 early region and the Rous sarcoma virus long terminal repeat) and cloned. The cell culture conditions are detailed in Supplementary Materials.

### 4.2. Pleural Fluid Samples

PF samples were collected at the Pleural Medicine Unit of the Hospital Universitari Arnau de Vilanova (Lleida, Spain). The samples were centrifuged to remove the cellular block. Informed consent for the acquisition and use of patient samples and clinical information was obtained from each patient. This project was approved by the Biosecurity Committee of IRBLleida (CEIC-1947). To guarantee the innocuity of the samples, PF supernatants were subjected to heat treatment at 60 °C for 30 min. Cell experiments were performed with 3 benign PF samples (B1–B3) and 12 MPEs (M1–M12). These PF samples were selected from a local biobank collection that included PF and plasma from approximately 400 patients in order to comprehensively represent the major etiologies of PE. Whole miRNA sequencing was performed with 6 benign PF samples (B1–B6) and 6 malignant PF samples (M2, M5, M6, M8, M9, and M12) to ensure that the groups were well matched. In the experiments, sample pairing was performed randomly, unless otherwise indicated. The diagnosis of MPE was made based on the presence of malignant cells in the PF or pleural biopsy specimens. Malignancy was also accepted in patients with a primary tumor and cytology-negative PF in whom other causes of pleural effusions had been reasonably excluded. The identification of benign pleural effusions relied on well-established clinical criteria [57].

**Figure 5:**
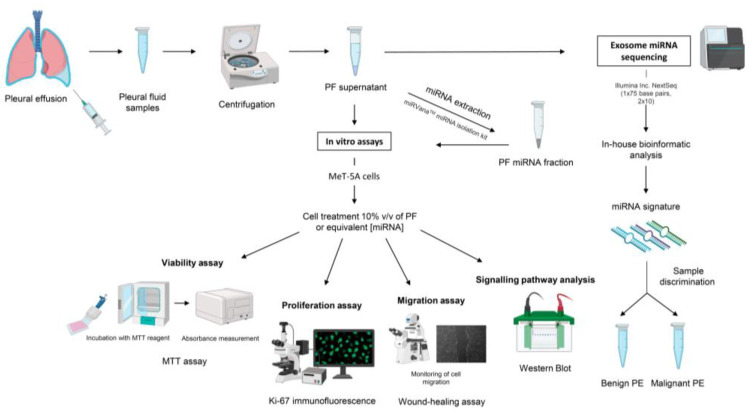
Schematic representation of the experimental procedures that were performed. Abbreviations: miRNA, microRNA; MTT, 3-(4,5-dimethylthiazol-2-yl)-2,5-diphenyltetrazolium bromide; PE, pleural effusion; PF, pleural fluid; *v*/*v*, volume/volume.

A schematic illustration of the methods that were utilized in this study is presented in Figure 5. All the experiments were performed on at least three independent biological replicates.

### 4.3. Total miRNA Isolation

miRNA was extracted from PF using the mirVana isolation kit (Thermo Fisher, Waltham, MA, USA) according to the manufacturer’s protocol. The miRNA content was determined with a NanoDrop 2000/2000c (Thermo Fisher, Waltham, MA, USA).

### 4.4. Functional Assays and Protein Expression Analysis by Western Blotting

After seeding, MeT-5A cells were treated with PF and subjected to several functional assays. Cell viability was determined with the 3-(4,5-dimethylthiazol-2-yl)-2,5-diphenyltetrazolium bromide (MTT) assay. Cell proliferation was assessed using Ki-67 immunofluorescence. Cell migration was determined with a wound healing assay. Finally, protein expression analysis was performed by Western blotting. The detailed protocols for these assays are described in Supplementary Materials [58,59,60].

### 4.5. Exosome-miRNA Sequencing

Next-generation sequencing (NGS) technology was performed to elucidate the miRNA profiles of PF samples B1, B2, B3, B4, B5, B6, M2, M5, M6, M8, M9, and M12. The detailed procedure can be found in the “Exosome-miRNA sequencing” section of Supplementary Materials.

### 4.6. Real Time-Quantitative Polymerase Chain Reaction

MeT-5A cells were treated with cell media supplemented with 10% of PF for 0, 24, 48, and 72 h. After each treatment, 1 µg RNA of MeT-5A cells was extracted using the Trizol^®^ reagent (Fisher Scientific, Waltham, MA, USA). One-step q-PCR kit (BioRad, Hercules, CA, USA) was used to convert total RNA into total DNA with CFX-Manager96 (BioRad, Hercules, CA, USA). Probes for *NCAD* and *VIM* were from Integrated DNA Technologies. All the conditions were normalized to 0 h. The value of 0 h was taken as 1.

### 4.7. Statistical Analysis

Statistical analysis was performed with Microsoft Office Excel 2019, Statistical Package for the Social Sciences Inc. (IBM SPSS Statistics 24, Chicago, IL, USA), and R software 4.2.1 [61]. Graphs were generated with GraphPad Prism v9.1.2. and R software 4.2.1. The significance of the proliferation and migration assay results was determined by unpaired two-tailed Student’s *t*-test. To control for false discovery rates (FDRs), the Benjamini–Hochberg procedure was applied. The discriminative miRNAs between benign effusions and MPEs were confirmed by the Boruta algorithm. Determination of the sorted predicted probabilities of malignancy was performed by a random forest analysis. A more detailed explanation of the statistical analysis can be found in Supplementary Materials [62].

## 5. Conclusions

We have shown that treatment with either malignant PF or miRNAs extracted from malignant PF enhances the migration of normal mesothelial MeT-5A cells in vitro. We also identified a three-miRNA signature that could be responsible, in part, for the development of MPE through host–tumor cell interaction in the tumor microenvironment. More importantly, this miRNA signature might be able to correctly classify MPEs with false-negative cytological examination results, thus potentially enhancing the sensitivity of standard diagnostic procedures.

## Figures and Tables

**Table 1:** Clinical characteristics of the study population.

Sample	Age (Years)	Sex	Diagnosis	Histological Subtype	PF Cytological Examination	Pleural Biopsy
B1	49	Male	Heart failure	NA	Negative	Negative
B2	75	Female	Heart failure	NA	ND	ND
B3	77	Male	Heart failure	NA	Negative	ND
B4	63	Female	Heart failure	NA	Negative	ND
B5	83	Male	Heart failure	NA	Negative	ND
B6	86	Female	Heart failure	NA	Negative	Negative
M1	50	Female	Breast cancer	Luminal B/HER2-	Positive	ND
M2	88	Female	Breast cancer	TNBC	Negative	ND
M3	70	Female	Breast cancer	Luminal B/HER2+	Positive	ND
M4	72	Male	NSCLC	Adenocarcinoma	Positive	ND
M5	65	Female	NSCLC	Adenocarcinoma	Positive	ND
M6	56	Female	NSCLC	Adenocarcinoma	Positive	ND
M7	69	Male	Colorectal cancer	Adenocarcinoma	Positive	ND
M8	60	Male	Colorectal cancer	Adenocarcinoma	Positive	ND
M9	84	Male	Colorectal cancer	Adenocarcinoma	Positive	Negative
M10	68	Male	Non-Hodgkin lymphoma	DLBCL	Negative *	ND
M11	90	Male	Non-Hodgkin lymphoma	DLBCL	Negative *	Negative
M12	83	Female	Non-Hodgkin lymphoma	Follicular large B-cell lymphoma	Negative *	ND

* The diagnosis of pleural malignancy was established by flow cytometric analysis of pleural fluid. DLBCL, diffuse large B-cell lymphoma; HER2, human epidermal growth factor receptor 2; NA, not applicable; ND, not done; NSCLC, non-small cell lung cancer; PF, pleural fluid; TNBC, triple negative breast cancer.

## Data Availability

The data that support the findings of this study are available from the corresponding author upon reasonable request.

## Supplementary Materials

The following supporting information can be downloaded at: https://www.mdpi.com/article/10.3390/ijms241814022/s1 and cited in ([59,60,61,62]).

## References

[1] Psallidas I., Kalomenidis I., Porcel J.M., Robinson B.W., Stathopoulos G.T. (2016). Malignant pleural effusion: From bench to bedside. Eur. Respir. Rev..

[2] Porcel J.M., Esquerda A., Vives M., Bielsa S. (2014). Etiology of pleural effusions: Analysis of more than 3000 consecutive thoracenteses. Arch. Bronconeumol..

[3] Massague J., Obenauf A.C. (2016). Metastatic colonization by circulating tumour cells. Nature.

[4] Stathopoulos G.T., Kalomenidis I. (2012). Malignant pleural effusion: Tumor-host interactions unleashed. Am. J. Respir. Crit. Care Med..

[5] Stathopoulos G.T., Zhu Z., Everhart M.B., Kalomenidis I., Lawson W.E., Bilaceroglu S., Peterson T.E., Mitchell D., Yull F.E., Light R.W. (2006). Nuclear factor-kappaB affects tumor progression in a mouse model of malignant pleural effusion. Am. J. Respir. Cell Mol. Biol..

[6] Stathopoulos G.T., Kollintza A., Moschos C., Psallidas I., Sherrill T.P., Pitsinos E.N., Vassiliou S., Karatza M., Papiris S.A., Graf D. (2007). Tumor necrosis factor-alpha promotes malignant pleural effusion. Cancer Res..

[7] Hooper C.E., Elvers K.T., Welsh G.I., Millar A.B., Maskell N.A. (2012). VEGF and sVEGFR-1 in malignant pleural effusions: Association with survival and pleurodesis outcomes. Lung Cancer.

[8] Thomas R., Cheah H.M., Creaney J., Turlach B.A., Lee Y.C. (2016). Longitudinal Measurement of Pleural Fluid Biochemistry and Cytokines in Malignant Pleural Effusions. Chest.

[9] Chen Y.M., Yang W.K., Whang-Peng J., Kuo B.I., Perng R.P. (1996). Elevation of interleukin-10 levels in malignant pleural effusion. Chest.

[10] Yeh H.H., Lai W.W., Chen H.H., Liu H.S., Su W.C. (2006). Autocrine IL-6-induced Stat3 activation contributes to the pathogenesis of lung adenocarcinoma and malignant pleural effusion. Oncogene.

[11] Calin G.A., Croce C.M. (2006). MicroRNA signatures in human cancers. Nat. Rev. Cancer.

[12] Calin G.A., Dumitru C.D., Shimizu M., Bichi R., Zupo S., Noch E., Aldler H., Rattan S., Keating M., Rai K. (2002). Frequent deletions and down-regulation of micro- RNA genes miR15 and miR16 at 13q14 in chronic lymphocytic leukemia. Proc. Natl. Acad. Sci. USA.

[13] Esquela-Kerscher A., Slack F.J. (2006). Oncomirs—microRNAs with a role in cancer. Nat. Rev. Cancer.

[14] Calin G.A., Liu C.G., Sevignani C., Ferracin M., Felli N., Dumitru C.D., Shimizu M., Cimmino A., Zupo S., Dono M. (2004). MicroRNA profiling reveals distinct signatures in B cell chronic lymphocytic leukemias. Proc. Natl. Acad. Sci. USA.

[15] Yanaihara N., Caplen N., Bowman E., Seike M., Kumamoto K., Yi M., Stephens R.M., Okamoto A., Yokota J., Tanaka T. (2006). Unique microRNA molecular profiles in lung cancer diagnosis and prognosis. Cancer Cell.

[16] Roldo C., Missiaglia E., Hagan J.P., Falconi M., Capelli P., Bersani S., Calin G.A., Volinia S., Liu C.G., Scarpa A. (2006). MicroRNA expression abnormalities in pancreatic endocrine and acinar tumors are associated with distinctive pathologic features and clinical behavior. J. Clin. Oncol..

[17] Sorolla M.A., Sorolla A., Parisi E., Salud A., Porcel J.M. (2021). Diving into the Pleural Fluid: Liquid Biopsy for Metastatic Malignant Pleural Effusions. Cancers.

[18] Zhai K., Shi X.Y., Yi F.S., Huang Z.Y., Wu X.Z., Dong S.F., Wang W., Wu M.T., Shi H.Z. (2020). IL-10 promotes malignant pleural effusion by regulating TH 1 response via an miR-7116-5p/GPR55/ERK pathway in mice. Eur. J. Immunol..

[19] Qian Q., Sun W., Zhu W., Liu Y., Ge A., Ma Y., Zhang Y., Zeng X., Huang M. (2017). The role of microRNA-93 regulating angiopoietin2 in the formation of malignant pleural effusion. Cancer Med..

[20] Lv M., Xu Y., Tang R., Ren J., Shen S., Chen Y., Liu B., Hou Y., Wang T. (2014). miR141-CXCL1-CXCR2 signaling-induced Treg recruitment regulates metastases and survival of non-small cell lung cancer. Mol. Cancer Ther..

[21] Porcel J.M. (2019). Diagnosis and characterization of malignant effusions through pleural fluid cytological examination. Curr. Opin. Pulm. Med..

[22] Vazquez F., Ramaswamy S., Nakamura N., Sellers W.R. (2000). Phosphorylation of the PTEN tail regulates protein stability and function. Mol. Cell Biol..

[23] Asciak R., Kanellakis N.I., Yao X., Abd Hamid M., Mercer R.M., Hassan M., Bedawi E.O., Dobson M., Fsadni P., Montefort S. (2021). Pleural Fluid Has Pro-Growth Biological Properties Which Enable Cancer Cell Proliferation. Front. Oncol..

[24] Cheah H.M., Lansley S.M., Varano Della Vergiliana J.F., Tan A.L., Thomas R., Leong S.L., Creaney J., Lee Y.C. (2017). Malignant pleural fluid from mesothelioma has potent biological activities. Respirology.

[25] Gartel A.L., Radhakrishnan S.K. (2005). Lost in transcription: p21 repression, mechanisms, and consequences. Cancer Res..

[26] Yin T., Wang G., He S., Shen G., Su C., Zhang Y., Wei X., Ye T., Li L., Yang S. (2016). Malignant Pleural Effusion and ascites Induce Epithelial-Mesenchymal Transition and Cancer Stem-like Cell Properties via the Vascular Endothelial Growth Factor (VEGF)/Phosphatidylinositol 3-Kinase (PI3K)/Akt/Mechanistic Target of Rapamycin (mTOR) Pathway. J. Biol. Chem..

[27] Gary Lee Y.C., Melkerneker D., Thompson P.J., Light R.W., Lane K.B. (2002). Transforming growth factor beta induces vascular endothelial growth factor elaboration from pleural mesothelial cells in vivo and in vitro. Am. J. Respir. Crit. Care Med..

[28] Maki Y., Nishimura Y., Toyooka S., Soh J., Tsukuda K., Shien K., Furukawa M., Muraoka T., Ueno T., Tanaka N. (2016). The proliferative effects of asbestos-exposed peripheral blood mononuclear cells on mesothelial cells. Oncol. Lett..

[29] Stathopoulos G.T., Sherrill T.P., Karabela S.P., Goleniewska K., Kalomenidis I., Roussos C., Fingleton B., Yull F.E., Peebles R.S., Blackwell T.S. (2010). Host-derived interleukin-5 promotes adenocarcinoma-induced malignant pleural effusion. Am. J. Respir. Crit. Care Med..

[30] Valadi H., Ekstrom K., Bossios A., Sjostrand M., Lee J.J., Lotvall J.O. (2007). Exosome-mediated transfer of mRNAs and microRNAs is a novel mechanism of genetic exchange between cells. Nat. Cell Biol..

[31] De Pascalis C., Etienne-Manneville S. (2017). Single and collective cell migration: The mechanics of adhesions. Mol. Biol. Cell.

[32] Sedgwick A.E., D’Souza-Schorey C. (2016). Wnt Signaling in Cell Motility and Invasion: Drawing Parallels between Development and Cancer. Cancers.

[33] Huang C., Jacobson K., Schaller M.D. (2004). MAP kinases and cell migration. J. Cell Sci..

[34] Wang Y., Xu Y.M., Zou Y.Q., Lin J., Huang B., Liu J., Li J., Zhang J., Yang W.M., Min Q.H. (2017). Identification of differential expressed PE exosomal miRNA in lung adenocarcinoma, tuberculosis, and other benign lesions. Medicine.

[35] Mitra A., Yoshida-Court K., Solley T.N., Mikkelson M., Yeung C.L.A., Nick A., Lu K., Klopp A.H. (2021). Extracellular vesicles derived from ascitic fluid enhance growth and migration of ovarian cancer cells. Sci. Rep..

[36] Wang H.Y., Liu Y.N., Wu S.G., Hsu C.L., Chang T.H., Tsai M.F., Lin Y.T., Shih J.Y. (2020). MiR-200c-3p suppression is associated with development of acquired resistance to epidermal growth factor receptor (EGFR) tyrosine kinase inhibitors in EGFR mutant non-small cell lung cancer via a mediating epithelial-to-mesenchymal transition (EMT) process. Cancer Biomark..

[37] Liu Y., Zhang Y., Li Q., Xu R., Huang N. (2022). MiR-200c-3p and miR-485-5p overexpression elevates cisplatin sensitivity and suppresses the malignant phenotypes of non-small cell lung cancer cells through targeting RRM2. Thorac. Cancer.

[38] Kang E., Jung S.C., Nam S.K., Park Y., Seo S.H., Park K.U., Oh H.K., Kim D.W., Kang S.B., Lee H.S. (2022). Tissue miR-200c-3p and circulating miR-1290 as potential prognostic biomarkers for colorectal cancer. Sci. Rep..

[39] Navarro-Manzano E., Luengo-Gil G., Gonzalez-Conejero R., Garcia-Garre E., Garcia-Martinez E., Garcia-Torralba E., Chaves-Benito A., Vicente V., Ayala de la Pena F. (2022). Prognostic and Predictive Effects of Tumor and Plasma miR-200c-3p in Locally Advanced and Metastatic Breast Cancer. Cancers.

[40] Nersisyan S., Gorbonos A., Makhonin A., Zhiyanov A., Shkurnikov M., Tonevitsky A. (2022). isomiRTar: A comprehensive portal of pan-cancer 5′-isomiR targeting. PeerJ.

[41] Wang Z., Zhao Z., Yang Y., Luo M., Zhang M., Wang X., Liu L., Hou N., Guo Q., Song T. (2018). MiR-99b-5p and miR-203a-3p Function as Tumor Suppressors by Targeting IGF-1R in Gastric Cancer. Sci. Rep..

[42] Qian Z., Gong L., Mou Y., Han Y., Zheng S. (2019). MicroRNA-203a-3p is a candidate tumor suppressor that targets thrombospondin 2 in colorectal carcinoma. Oncol. Rep..

[43] Xu J.Z., Shao C.C., Wang X.J., Zhao X., Chen J.Q., Ouyang Y.X., Feng J., Zhang F., Huang W.H., Ying Q. (2019). circTADA2As suppress breast cancer progression and metastasis via targeting miR-203a-3p/SOCS3 axis. Cell Death Dis..

[44] Xu H., Lan Q., Huang Y., Zhang Y., Zeng Y., Su P., Chu Z., Lai W., Chu Z. (2021). The mechanisms of colorectal cancer cell mesenchymal-epithelial transition induced by hepatocyte exosome-derived miR-203a-3p. BMC Cancer.

[45] Chen L., Gao H., Liang J., Qiao J., Duan J., Shi H., Zhen T., Li H., Zhang F., Zhu Z. (2018). miR-203a-3p promotes colorectal cancer proliferation and migration by targeting PDE4D. Am. J. Cancer Res..

[46] Aakko S., Straume A.H., Birkeland E.E., Chen P., Qiao X., Lonning P.E., Kallio M.J. (2019). MYC-Induced miR-203b-3p and miR-203a-3p Control Bcl-xL Expression and Paclitaxel Sensitivity in Tumor Cells. Transl. Oncol..

[47] Shu S., Wu H.J., Ge J.Y., Zeid R., Harris I.S., Jovanovic B., Murphy K., Wang B., Qiu X., Endress J.E. (2020). Synthetic Lethal and Resistance Interactions with BET Bromodomain Inhibitors in Triple-Negative Breast Cancer. Mol. Cell.

[48] Han B., Molins L., He Y., Vinolas N., Sanchez-Lorente D., Boada M., Guirao A., Diaz T., Martinez D., Ramirez J. (2022). Characterization of the MicroRNA Cargo of Extracellular Vesicles Isolated from a Pulmonary Tumor-Draining Vein Identifies miR-203a-3p as a Relapse Biomarker for Resected Non-Small Cell Lung Cancer. Int. J. Mol. Sci..

[49] Li J.Z., Li J., Wang H.Q., Li X., Wen B., Wang Y.J. (2017). MiR-141-3p promotes prostate cancer cell proliferation through inhibiting kruppel-like factor-9 expression. Biochem. Biophys. Res. Commun..

[50] Ye Y., Li S.L., Ma Y.Y., Diao Y.J., Yang L., Su M.Q., Li Z., Ji Y., Wang J., Lei L. (2017). Exosomal miR-141-3p regulates osteoblast activity to promote the osteoblastic metastasis of prostate cancer. Oncotarget.

[51] Moon S.U., Park Y., Park M.G., Song S.K., Jeong S.H., Lee Y.S., Heo H.J., Jung W.Y., Kim S. (2019). Theragnosis by a miR-141-3p molecular beacon: Simultaneous detection and sensitization of 5-fluorouracil resistant colorectal cancer cells through the activation of the TRIM13-associated apoptotic pathway. Chem. Commun..

[52] Masoumi-Dehghi S., Babashah S., Sadeghizadeh M. (2020). microRNA-141-3p-containing small extracellular vesicles derived from epithelial ovarian cancer cells promote endothelial cell angiogenesis through activating the JAK/STAT3 and NF-kappaB signaling pathways. J. Cell Commun. Signal.

[53] Xiao Y., Liu G., Sun Y., Gao Y., Ouyang X., Chang C., Gong L., Yeh S. (2020). Targeting the estrogen receptor alpha (ERalpha)-mediated circ-SMG1.72/miR-141-3p/Gelsolin signaling to better suppress the HCC cell invasion. Oncogene.

[54] Dasgupta P., Kulkarni P., Majid S., Hashimoto Y., Shiina M., Shahryari V., Bhat N.S., Tabatabai L., Yamamura S., Saini S. (2020). LncRNA CDKN2B-AS1/miR-141/cyclin D network regulates tumor progression and metastasis of renal cell carcinoma. Cell Death Dis..

[55] Phatak P., Noe M., Asrani K., Chesnick I.E., Greenwald B.D., Donahue J.M. (2021). MicroRNA-141-3p regulates cellular proliferation, migration, and invasion in esophageal cancer by targeting tuberous sclerosis complex 1. Mol. Carcinog..

[56] Chao F., Song Z., Wang S., Ma Z., Zhuo Z., Meng T., Xu G., Chen G. (2021). Novel circular RNA circSOBP governs amoeboid migration through the regulation of the miR-141-3p/MYPT1/p-MLC2 axis in prostate cancer. Clin. Transl. Med..

[57] Porcel J.M., Light R.W. (2013). Pleural effusions. Dis. Mon..

[58] Filetti V., Lombardo C., Loreto C., Dounias G., Bracci M., Matera S., Rapisarda L., Rapisarda V., Ledda C., Vitale E. (2023). Small RNA-Seq Transcriptome Profiling of Mesothelial and Mesothelioma Cell Lines Revealed microRNA Dysregulation after Exposure to Asbestos-like Fibers. Biomedicines.

[59] Yang D., Chen C., Xia H., Chen J., Yu M. (2023). Characteristics of transcription profile, adhesion and migration of SETD2-loss Met-5A mesothelial cells exposed with crocidolite. J. Appl. Toxicol..

[60] Dell’Anno I., Barbarino M., Barone E., Giordano  A., Luzzi L., Bottaro M., Migliore L., Agostini S., Melani A., Melaiu O. (2020). Catalano C, Cipollini M, Silvestri R, Corrado A, Gemignani F, Landi S. EIF4G1 and RAN as Possible Drivers for Malignant Pleural Mesothelioma. Int. J. Mol. Sci..

[61] R Core Team (2022). A Language and Environment for Statistical Computing.

[62] Dweep H., Sticht C., Pandey P., Gretz N. (2011). miRWalk—database: Prediction of possible miRNA binding sites by "walking" the genes of three genomes. J. Biomed. Inform..

