# Supplementary material for: MicroRNAs Present in Malignant Pleural Fluid Increase the Migration of Normal Mesothelial Cells In Vitro and May Help Discriminate between Benign and Malignant Effusions
Source: Int J Mol Sci. 2023 Sep 13;24(18):14022. doi: 10.3390/ijms241814022 (PMC10531386; doi:10.3390/ijms241814022)
Supplement: Supplementary file 1 [file ijms-24-14022-s001.zip › Supplementary Figure Legends.pdf]

Supplementary Figure Legends

Figure S1. Effects of the miRNA fraction of malignant pleural fluid on the viability and proliferation of normal mesothelial MeT-5A cells.

(a) Viability of MeT-5A cells exposed to different extracted miRNAs for 24 h. The line indicates 100% viability.

(b) Representative images of Ki-67 immunofluorescence (red) 24 h after treatment with miRNAs. Each image is shown with the corresponding nuclear staining (Hoechst). Scale bar indicates 200 μm. At the bottom, a graph of Ki-67 quantification shows the percentage of Ki-67-positive cells.

The data are represented as the mean ± SD (n = 3). Differences were considered significant at $p < 0.05$ (*), $p < 0.01$ (**), and $p < 0.001$ (***), as determined by two-tailed Student's t test.

Abbreviations: B, benign; M, malignant; NSCLC, non-small cell lung cancer; UN, untreated condition (control).

Figure S2. The malignant pleural fluid M9 increases the expression of the mesenchymal markers N-Cadherin and Vimentin in MeT-5A cells.

Left: graph of the relative expression of N-Cadherin and Vimentin obtained by quantitative real-time polymerase chain reaction (qRT-PCR) of MeT-5A cell lysates treated with media supplemented with 10% of benign pleural fluid B2.

Right: graph corresponding to MeT-5A cell lysates treated with media supplemented with 10% of malignant pleural fluid M9.

Abbreviations: B, benign; *ECAD*, E-Cadherin; h, hours; M, malignant; mRNA, messenger ribonucleic acid; *VIM*, Vimentin.

Figure S3. Effects of pleural fluid on the phosphorylation of different proteins that participate in cell survival- and proliferation-related signaling pathways.

Graphs showing the quantified band intensities from the western blots in Fig. 4; band intensities were determined using Image Lab 6.1 software (Bio-Rad). Phosphorylated/nonphosphorylated ratios were calculated for each protein, considering the volume intensity parameter and its normalization to tubulin.

Abbreviations: B, benign; M, malignant.

Figure S4. Effects of miRNAs on the phosphorylation of different proteins that participate in cell survival- and proliferation-related signaling pathways.

Left: western blot images showing whole protein lysates of MeT-5A cells treated with PF samples B2 and M12 for 0, 0.5, 3, 8, 24 and 48 h. The antibodies that were used recognized tensin homolog serine 380 (P-PTEN S380), phospho-AKT serine 473 (P-AKT S473), phospho-AKT threonine 308 (P-AKT T308), pan AKT, phospho-p42/44 MAPK threonine 202/tyrosine 204 (P-p42/44 MAPK T202/Y204), p42/44 MAPK, and p21. Tubulin was used as the control. Right: graphs showing the quantified band intensities from the western blots in Fig. 4; band intensities were determined using Image Lab 6.1 software (Bio-Rad). Phosphorylated/nonphosphorylated ratios were calculated for each protein, considering the volume intensity parameter and its normalization to tubulin.

Abbreviations: B, benign; M, malignant; PF, pleural fluid.

Figure S5. Diagram that represents the AKT and MAPK signaling pathways and the main participating proteins. In bold, the proteins studied by Western Blot in this study. The arrows pointing up and down represent the observed overexpression or under expression of the studied proteins, respectively.

Abbreviations: AKT, protein kinase B; ERK, extracellular signal-regulated kinase; MAPK, mitogen-activated protein kinase; MEK, mitogen-activated protein kinase; mTORC1, mammalian target of rapamycin complex 1; PIP2, phosphatidylinositol 4,5-bisphosphate; PIP3, phosphatidylinositol (3,4,5)-trisphosphate; PI3K, phosphatidylinositol-3-kinase;

PTEN, phosphatase and tensin homolog; RAS, rat sarcoma; RAF, rapidly accelerated fibrosarcoma; Rheb, ras homolog enriched in brain S, serine; T, threonine.
