# Supplementary material for: MicroRNAs Present in Malignant Pleural Fluid Increase the Migration of Normal Mesothelial Cells In Vitro and May Help Discriminate between Benign and Malignant Effusions
Source: Int J Mol Sci. 2023 Sep 13;24(18):14022. doi: 10.3390/ijms241814022 (PMC10531386; doi:10.3390/ijms241814022)
Supplement: Supplementary file 1 [file ijms-24-14022-s001.zip › Supplementary Material IJMS.pdf]

Supplementary Material

1. Materials

1.1 Cell culture conditions

MeT-5A cells were cultured at 37°C in a 5% CO2 incubator with a humidified atmosphere. This cell line was cultured in Medium 199 supplemented with 1.5 g/L sodium bicarbonate, 10% fetal bovine serum (FBS), 3.3 nM epidermal growth factor, 1% penicillin–streptomycin, 400 nM hydrocortisone, 870 nM zinc-free bovine insulin, 20 mM HEPES and a buffer solution of Earle's salts (according to the manufacturer's instructions).

2. Methods

2.1 Cell viability assay

MeT-5A viability was determined by the 3-(4,5-dimethylthiazol-2-yl)-2,5-diphenyltetrazolium bromide (MTT) assay (Sigma–Aldrich). Cells were plated in a 96-well culture plate (10,000 cells/well). After seeding, the cells were incubated at 37°C in 5% CO2 for at least 24 hours (h) [1,2]. The next day, the cell medium was replaced with fresh medium containing 10% PF in volume or an equivalent miRNA concentration. After 24 h of treatment, 100 µl of 0.5 mg/ml MTT solution was added to each well. Ninety minutes later, the medium was removed, 50 µl of dimethyl sulfoxide (DMSO) reagent (Sigma–Aldrich) was added to the wells, and the absorbance was quantified at 575 nm using an EPOCH spectrophotometer (BioTek). The viability of untreated cells was considered 100% cell viability, and the percentages of cell viability of the remaining conditions were normalized to that of the untreated condition.

2.2 Cell proliferation assay

For the proliferation assays, MeT-5A cells were plated in a 96-well culture plate (10,000 cells/well). After seeding, the cells were incubated at 37°C in 5% CO2 for at least 24 h [1,2]. The cells were then treated with medium containing 10% PF or an equivalent miRNA content for 24 h. The cells were then fixed with 4% paraformaldehyde diluted in phosphate-buffered saline (PBS), permeabilized

and blocked with 0.3% Triton X-100 and 5% normal goat serum (NGS) in PBS. An anti-Ki-67 mouse monoclonal primary antibody (A-11032, Invitrogen, 1:500) was diluted 1:500 in 1% bovine serum albumin and 0.3% Triton-X100 in PBS, and then, it was added to the cells and incubated overnight at 4°C. The cells were washed three times with PBS and then incubated with a goat anti-mouse Alexa Fluor 594-conjugated secondary antibody diluted in the same solution at room temperature for 1 h. The cells were again washed three times with PBS, incubated with Hoechst 62249 (Thermo Fisher) at 1:10,000 in PBS for 15 min at room temperature in the dark, and washed three more times. Fluorescence imaging was performed using a fluorescence microscope (NIKON ECLIPSE TS2R-FL). The percentage of Ki-67-positive cells (red) versus total cells (blue) was assessed in several fields of view. The results were normalized to those of untreated cells.

2.3 Cell migration assay

For the migration assays, 200,000 cells/well were seeded in a 6-well plate. After seeding, the cells were allowed to attach for 24 h. Then, the old medium was replaced with fresh medium supplemented with 10% PF or equivalent amounts of miRNAs. Immediately after that step, the center of the cell monolayer was scraped with a sterile micropipette tip to create a scratch or wound of constant width (time point 24 h). Wound healing was monitored and photographed with an EVOS FL Microscope (Invitrogen AMF4300) at 0, 24, 48, 72 and 96 h after cell seeding [2,3]. To quantify cell migration, pictures of the initial wounded monolayers were compared to pictures taken at the different timepoints, and the percentage of the cell-free area was quantified using ImageJ software (NIH, USA).

2.4 Western blotting

Cells were plated in 6-well plates at a density of 200,000 cells/well in 2 mL of 199 cell medium. After seeding, the cells were incubated at 37°C in 5% CO2 for 72 h. Then, the old media was replaced with fresh media supplemented with 10% PF or the corresponding isolated miRNAs. An untreated condition (0 h) was established in each plate. The cells were washed twice with cold PBS and lysed with cold protein lysis buffer (2% sodium dodecyl sulfate [SDS], 125 mM Tris-HCl, pH 6.8) at different time points: 0, 0.5, 3, 8, 24 and 48 h after treatment. The samples were sonicated for 20 s at 10 mA, and the

protein concentrations were quantified with the Detergent Compatible Protein Assay (Bio-Rad). Equal amounts of protein (15 μg) were mixed with 5X loading buffer. The protein samples were denatured using a thermal block at 95°C for 5 min and loaded into a polyacrylamide gel. The proteins were separated by electrophoresis at 100 V and transferred to polyvinylidene difluoride (PVDF) membranes (Bio-Rad) using a semidry system (Bio-Rad) for 1 h at 60 mA/membrane. The membranes were incubated with 5% nonfat milk in TBST (20 mM Tris-HCl, pH 7.4, 150 mM NaCl, and 0.1% Tween-20) to block nonspecific binding. The membranes were incubated overnight at 4°C with primary antibody diluted in 3% BSA and 0.02% sodium azide. We examined the phosphorylation of key residues and the expression of other proteins in the phosphatidylinositol 3-kinase (PI3K)/AKT/PKB signaling pathway and the mitogen-activated protein kinase (MAPK)/extracellular signal-regulated kinase (ERK) pathway: phospho-phosphatase and tensin homolog serine 380 (P-PTEN S380), phospho-AKT serine 473 (P-AKT S473), phospho-AKT threonine 308 (P-AKT T308), phospho-p42/44 MAPK threonine 202/tyrosine 204 (P-p42/44 MAPK T202/Y204), pan AKT, p42/44 MAPK and p21, which is a negative regulator of cell cycle progression. All these antibodies were obtained from Cell Signaling Technology (Danvers, MA, USA). The next day, the membranes were first incubated with the corresponding secondary antibody conjugated with horseradish peroxidase (HRP) at a dilution of 1:10,000 in 2% skim milk in TBS and then with the Chemiluminescent HRP Substrate Immobilon® Western (Millipore). The chemiluminescent signals were captured and quantified using Image Lab Software (Bio-Rad, Version 6.1). Tubulin (Sigma–Aldrich) was used as the loading control.

2.5 Exosome-miRNA sequencing

All the procedures were carried out at the Qiagen headquarters in Germany. An exosome isolation step was required before RNA isolation. RNA was isolated from 8 ml of each PF sample using exoRNeasy Maxi (Qiagen) according to the manufacturer's instructions. To ensure the quality of the extraction for sequencing, different synthetic miRNA (Spike-ins) concentrations were added to the sample before total RNA isolation. Library preparation was performed using the QIAseq miRNA Library Kit (QIAGEN). Library preparation was quality controlled using capillary electrophoresis

(Tape D1000). Based on the quality of the inserts and the concentration measurements, the libraries were pooled in equimolar ratios. The library pools were quantified using qPCR. The library pools were then sequenced on a NextSeq (Illumina Inc.) instrument according to the manufacturer's instructions (1 x 75 base pair, 2 x 10). Raw data were demultiplexed, and FASTQ files for each sample were generated using bcl2fastq2 software (Illumina Inc.). All the primary analyses were carried out using CLC Genomics Server 21.0.4. The workflow "QIAseq miRNA Quantification" of the CLC Genomics Server with standard parameters was used to map the reads to miRBase version 22. The 'Empirical analysis of DGE' algorithm of the CLC Genomics Workbench 21.0.4 was used for differential expression analysis with default settings. Finally, a report of data quality and simple bioinformatic analysis was delivered.

2.6 Statistical analyses

For the viability assay, the data were normalized to the average absorbance of the untreated cells, which were considered to represent 100% viability. For the proliferation assay, the data were normalized to the average proliferation of the untreated cells, which were similarly considered to indicate 100% proliferation. For the migration assay, the data were normalized to the 0 h time point. All the distributions were normal, and each average distribution was compared with the others using unpaired two-tailed Student's t test.

The miRNA sequencing data that were obtained were subjected to a comprehensive statistical and bioinformatic analysis. First, data were preprocessed by removing miRNAs with low expression levels and applying normalization for composition bias. Specifically, only miRNAs that were expressed at more than 1.35 counts per million (equivalent to 10-15 counts for the library sizes in our data) in at least 6 samples were retained (528 miRNAs). To investigate for differential expression, data were fitted to a negative binomial generalized log-linear model with the effect of sample type, and the Benjamini–Hochberg p value multiple testing adjustment method was applied to control for false discovery rate (FDR). Log ratio - log mean (MA), volcano plots, and a heatmap were built with FDR < 0.05 or 0.1. The discriminative miRNAs between samples were confirmed by the Boruta algorithm. A recursive

partitioning classification tree was built using the previously confirmed relevant miRNA by the Boruta algorithm and a minimum node size of 3. A Gardner-Altman two-group estimation plot was built on the out-of-bag predicted probabilities of a random forest analysis with the selected miRNAs. This plot shows these predicted probabilities of malignancy and their unpaired mean difference (M (malignant) minus B [benign]). The mean difference and the 95% confidence interval are displayed as a point estimate and vertical bar, respectively, using the sample density distribution calculated from a bias-corrected and accelerated bootstrap analysis of 5000 resamples. Finally, the resulting significantly expressed miRNAs (FDR < 0.05) were subjected to Gene Ontology (GO) analysis to screen relevant biological processes using the miRWalk v3 software (http://mirwalk.umm.uni-heidelberg.de/) [4] and its tool TargetScan.

References

1. Filetti V, Lombardo C, Loreto C, Dounias G, Bracci M, Matera S, Rapisarda L, Rapisarda V, Ledda C, Vitale E. Small RNA-Seq Transcriptome Profiling of Mesothelial and Mesothelioma Cell Lines Revealed microRNA Dysregulation after Exposure to Asbestos-like Fibers. Biomedicines. 2023 Feb 13;11(2):538. doi: 10.3390/biomedicines11020538.

2. Yang D, Chen C, Xia H, Chen J, Yu M. Characteristics of transcription profile, adhesion and migration of SETD2-loss Met-5A mesothelial cells exposed with crocidolite. J Appl Toxicol. 2023 May 5. doi: 10.1002/jat.4493.

3. Dell'Anno I, Barbarino M, Barone E, Giordano A, Luzzi L, Bottaro M, Migliore L, Agostini S, Melani A, Melaiu O, Catalano C, Cipollini M, Silvestri R, Corrado A, Gemignani F, Landi S. *EIF4G1* and *RAN* as Possible Drivers for Malignant Pleural Mesothelioma. Int J Mol Sci. 2020 Jul 9;21(14):4856. doi: 10.3390/ijms21144856.

4. Dweep H, Sticht C, Pandey P, Gretz N. miRWalk--database: prediction of possible miRNA binding sites by "walking" the genes of three genomes. J Biomed Inform. 2011 Oct;44(5):839-47. doi: 10.1016/j.jbi.2011.05.002.
