# Supplementary figures and images for: MicroRNAs Present in Malignant Pleural Fluid Increase the Migration of Normal Mesothelial Cells In Vitro and May Help Discriminate between Benign and Malignant Effusions
Source: Int J Mol Sci. 2023 Sep 13;24(18):14022. doi: 10.3390/ijms241814022 (PMC10531386; doi:10.3390/ijms241814022)

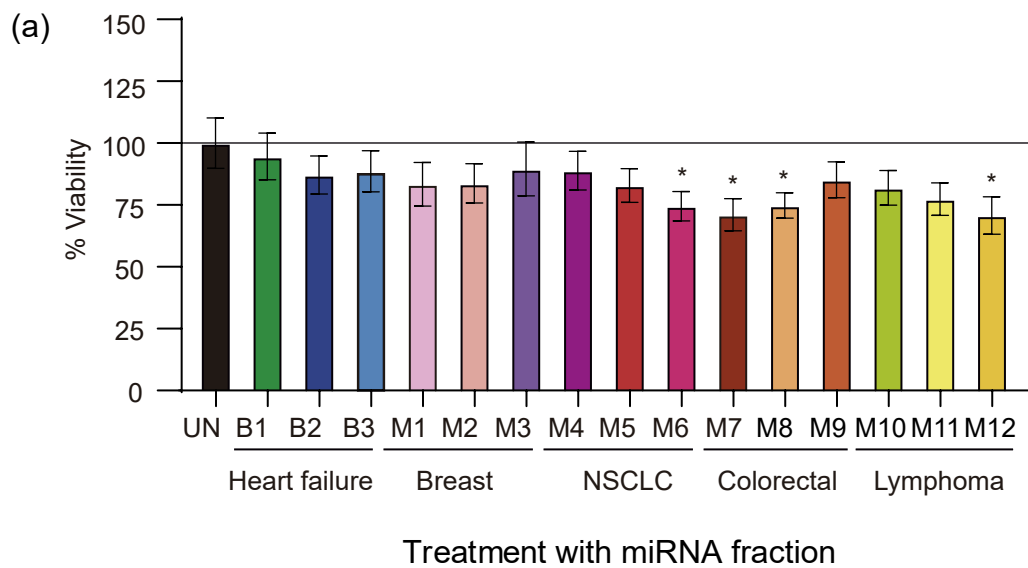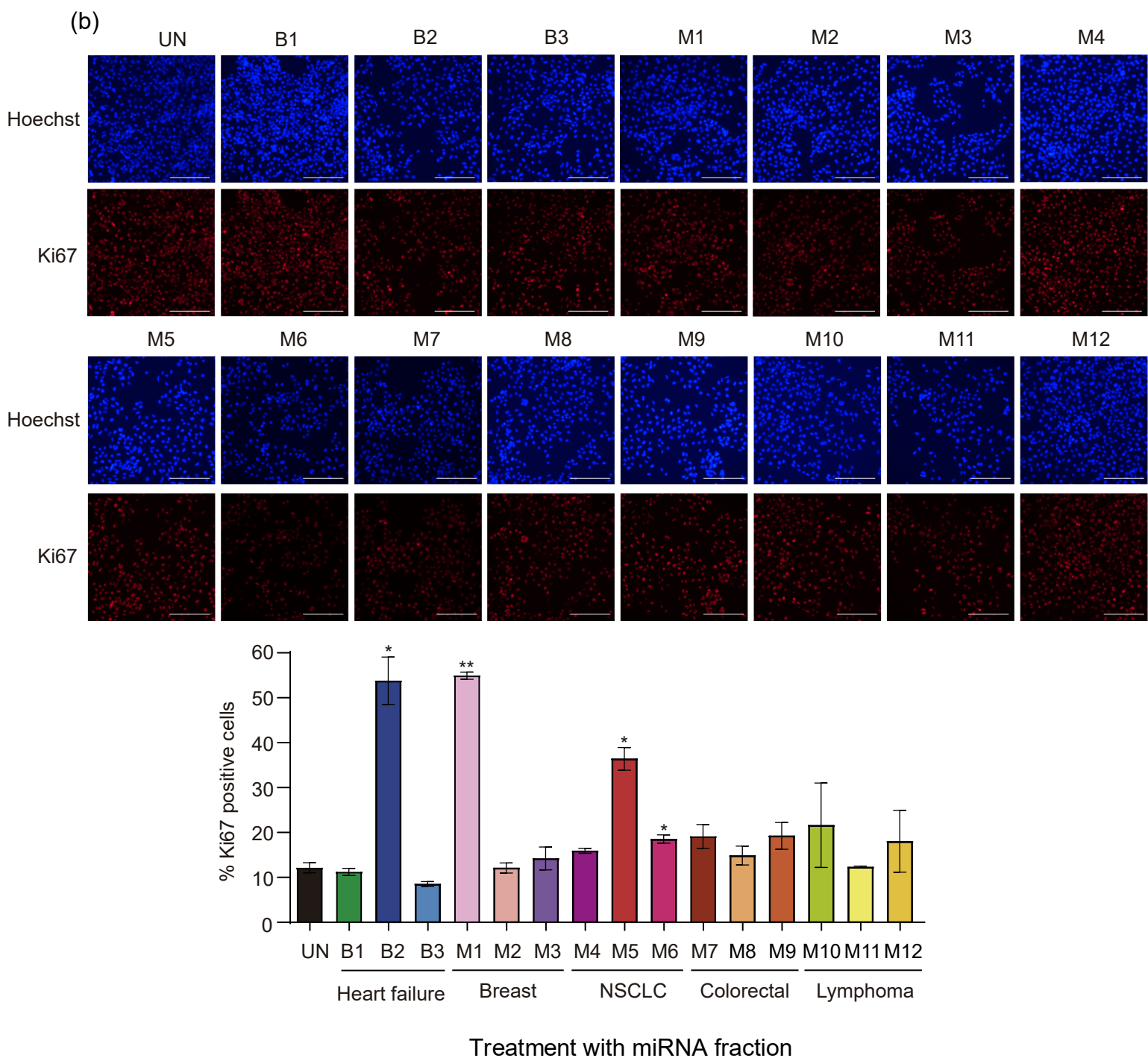

Supplement: Supplementary file 1 [file ijms-24-14022-s001.zip › Supplementary Figure 1.pdf]

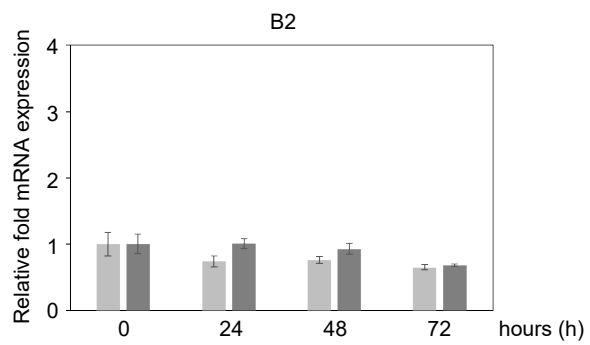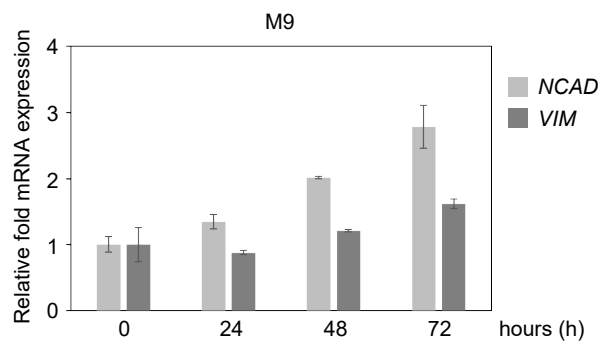

Supplement: Supplementary file 1 [file ijms-24-14022-s001.zip › Supplementary Figure 2.pdf]

**B1 versus M5**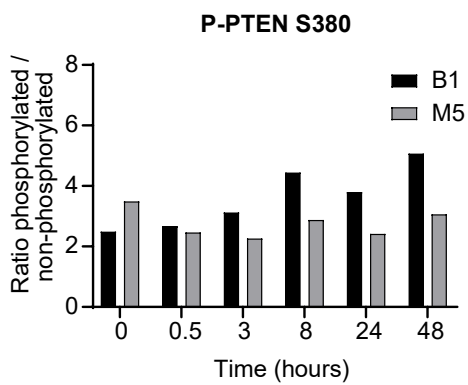**B2 versus M12**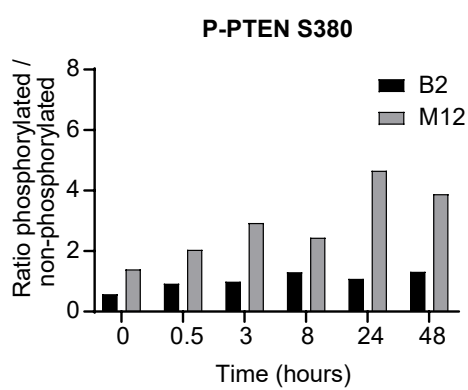**B3 versus M9**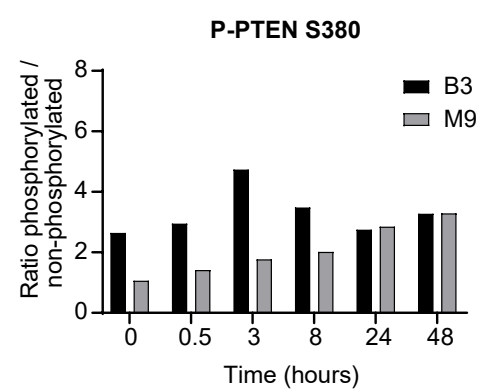**P-AKT S473**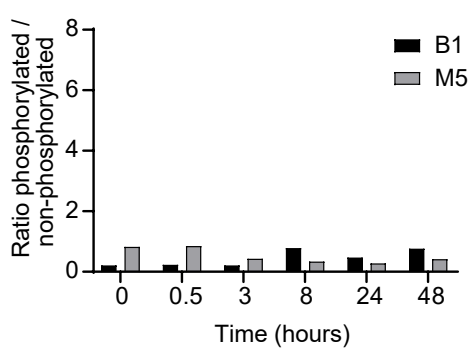**P-AKT S473**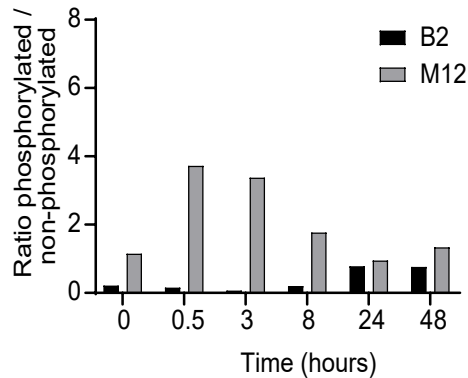**P-AKT S473**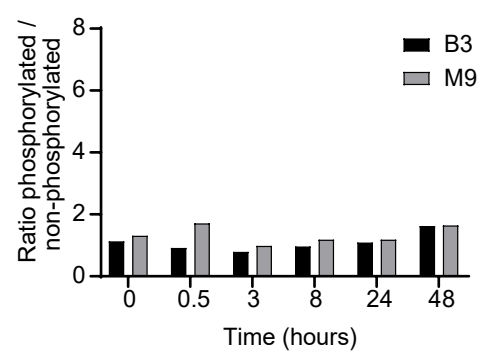**P-AKT T308**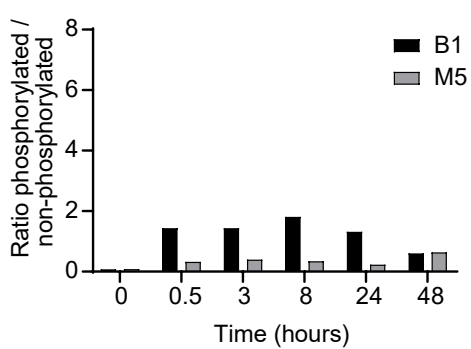**P-AKT T308**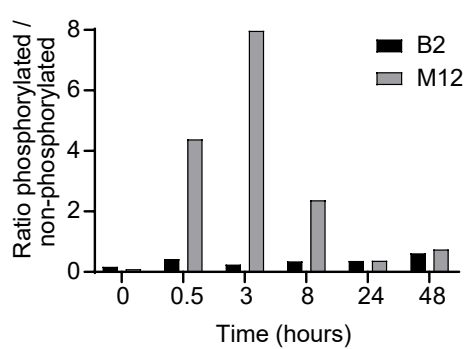**P-AKT T308**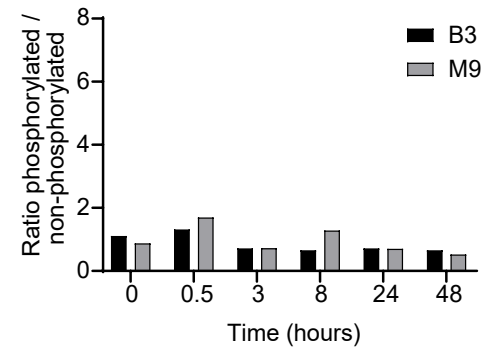**P-MAPK T202/Y204**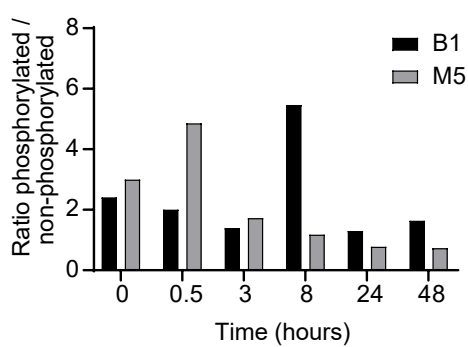**P-MAPK T202/Y204**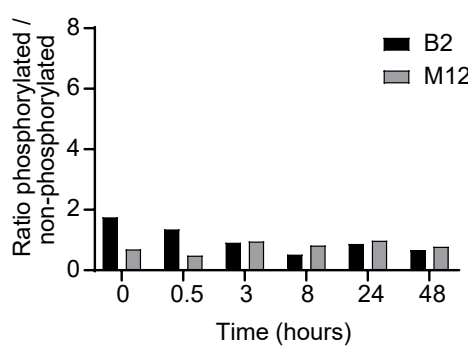**P-MAPK T202/Y204**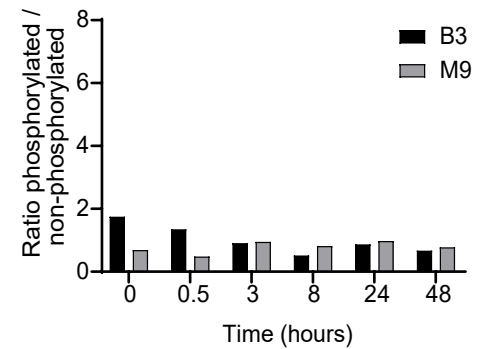

Supplement: Supplementary file 1 [file ijms-24-14022-s001.zip › Supplementary Figure 3.pdf]

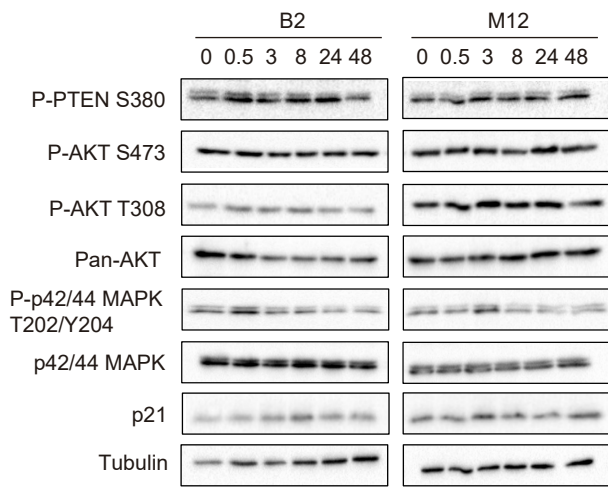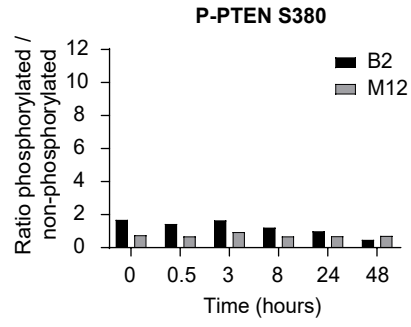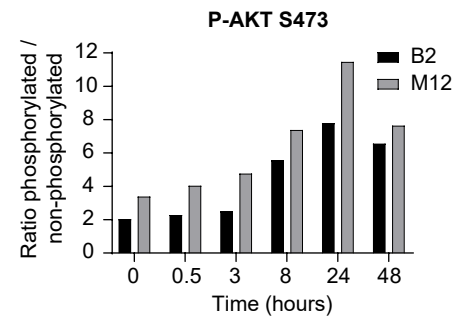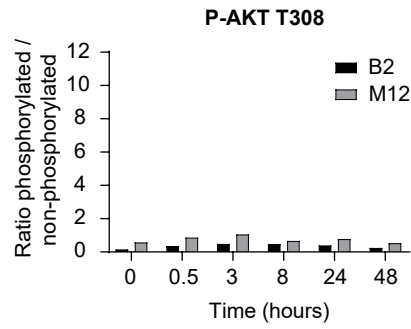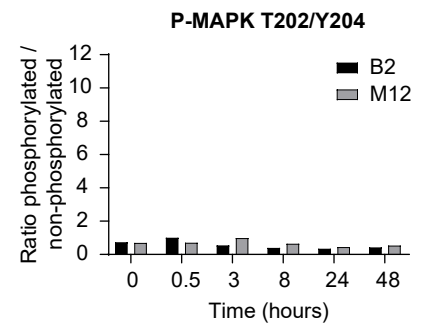

Supplement: Supplementary file 1 [file ijms-24-14022-s001.zip › Supplementary Figure 4.pdf]

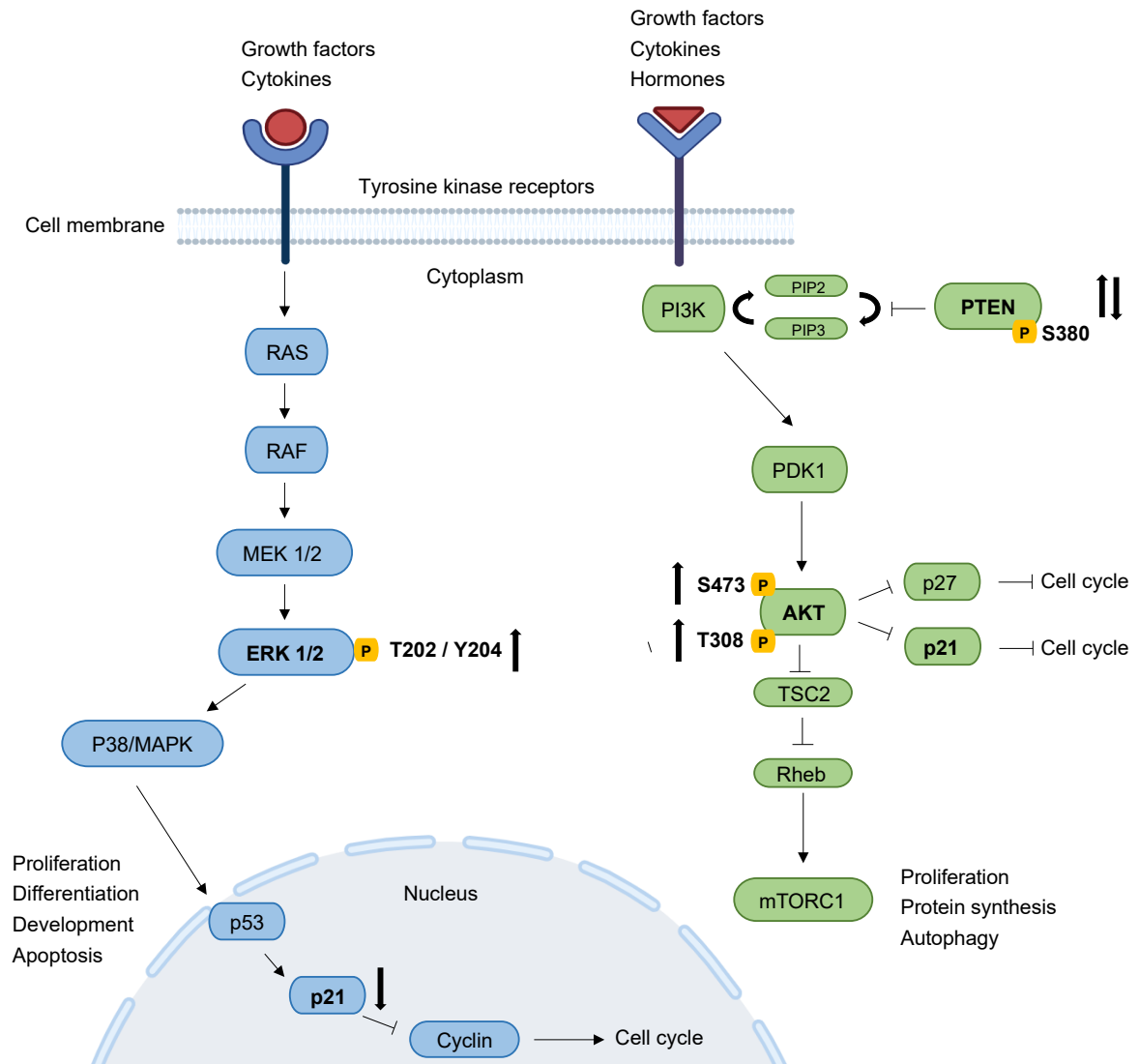

Supplement: Supplementary file 1 [file ijms-24-14022-s001.zip › Supplementary Figure 5.pdf]
